# Clonal dissemination of KPC-2 producing *Klebsiella pneumoniae* ST11 clone with high prevalence of *oqxAB* and *rmtB* in a tertiary hospital in China: results from a 3-year period

**DOI:** 10.1186/s12941-015-0109-x

**Published:** 2016-01-19

**Authors:** Li Cheng, Xiao-Li Cao, Zhi-Feng Zhang, Ming-zhe Ning, Xue-Jing Xu, Wanqing Zhou, Jun-Hao Chen, Jin-hua Zhang, Han Shen, Kui Zhang

**Affiliations:** Department of Laboratory Medicine, Nanjing Drum Tower Hospital, The Affiliated Hospital of Nanjing University Medical School, Zhongshan Road, 321#, Gulou District, Nanjing, Jiangsu People’s Republic of China

**Keywords:** KPC-2, ST11, OqxAB, MLST, *Klebsiella pneumonia*e

## Abstract

**Background:**

Carbapenemase-producing *Klebsiella pneumoniae* (CPKP) *strains* have emerged as a major problem for healthcare systems. The aim of this study was to determine the circulating clones and analyze the clinical and molecular characteristics of CPKP in our hospital.

**Methods:**

A total of 74 carbapenemase producers collected from our hospital from 2012 to 2014 were analyzed for the prevalence of extended-spectrum β-lactamase (ESBLs), plasmid-mediated quinolone resistance genes (PMQRs), exogenously acquired 16S rRNA methyltransferase (16S-RMTase), and plasmid-mediated AmpC enzyme (pAmpCs) by PCR and DNA sequencing. The sequence types (STs) of the carbapenemase producers were analyzed by multi-locus sequence typing (MLST). And Pulsed-field gel electrophoresis (PFGE) was performed to investigate the genetic relationship of KPC-2 producing strains. Clinical data were retrieved from the medical records.

**Results:**

KPC-2 (n = 72) was the predominant enzyme followed by NDM-1 (n = 2); The genes *bla*CTX-M, *bla*SHV, *bla*TEM-1, *bla*DHA-1, *rmtB*, *armA*, *oqxA*, *oqxB*, and *qnrB* were present in 29 (39.2 %), 27 (36.5 %), 46 (62.2 %), 2 (2.7 %), 25 (33.8 %), 1 (1.4 %), 60 (81.1 %) and 56 (75.7 %), 6 (8.1 %) isolates, respectively. MLST analysis revealed 10 different STs. The most dominant ST was ST11 (78.4 %, 58/74), followed by ST15 (8.1 %, 6/74). PFGE patterns of the KPC-2 producing *K. pneumoniae* isolates exhibited clonal dissemination of ST11 and ST15 clones as well as a genetic diversity of the remaining strains.

**Conclusion:**

The intra- and inter-hospital cross-transmission of KPC-2-producing *K*. *pneumoniae* ST11 co-carrying *oqxAB* and *rmtB* in our hospital strongly suggested that rapid identification of colonized or infected patients and screening of carriers is quite necessary to prevent a scenario of endemicity.

## Background

*Klebsiella pneumoniae* are part of the human gut microbiota and frequently cause infections from mild urinary and gastrointestinal infections to severe bacteremia and pneumonia with a high rate of mortality and morbidity [1]. An increasing emergence of carbapenem resistance among *K. pneumoniae* isolates worldwide has limited the therapeutic options for treatment of these infections [2].

Carbapenem resistance in *K. pneumoniae* is frequently caused by the predominant production of KPC carbapenemase worldwide [3], the production of OXA-48-like carbapenemase is currently spreading most rapidly in many European countries with IMP and VIM being occasionally detected [4]. And transmission of *bla*NDM are potentially global health issues [5]. In addition, Carriage of *bla*_ESBL_ and/or *bla*_AmpC_ in combination with active efflux or porin loss has also been shown to be responsible for carbapenem-resistance in *Enterobacteriaceae* [6].

Carbapenem resistant *K. pneumoniae* (CRKP) is reported to be caused either by epidemic clones or by the horizontal dissemination of mobile elements such as plasmids and insertion sequences. In Europe and American countries, KPC-producing ST258 *K. pneumoniae* can undeniably be regarded as one of the most successful multidrug-resistant nosocomial pathogens [7]. And the international high-risk clone of *K. pneumoniae* ST11 is frequently reported as a successful pathogen at infections in Asian and European countries because of the association of important co-resistance and virulence factors [8]. And the quick expansion and evolution of this multi-resistant and virulent clone may lead to an evolving crisis of global dimensions [9].

In recent years, CRKP has been emerging with an increasing frequency worldwide [2]. All these isolates were resistant to currently available antimicrobial agents except tigecycline. Extended-spectrum β-lactamase (ESBLs), exogenously acquired 16S rRNA methyltransferase (16S-RMTase) genes, and plasmid-mediated AmpC enzyme (pAmpCs) has been frequently detected among the clinical multidrug-resistant *Enterobacteriaceae* [10]. In addition, the prevalence of PMQR genes has been reported to be associated with the increasing quinolone resistance development in *Enterobacteriaceae* which involved an increase in the diversity of the PMQR genes [11]. Nevertheless, data on the distribution of these resistant determinants among carbapenemase-producing *K. pneumoniae* (CPKP) isolates collected during a long time period is still limited, and the dissemination of the internationally predominant CPKP isolate is still unclear.

The aim of the present study was to determine the circulating clones of CPKP and our study focused on a hospital-wide screening of ESBLs, 16S-RMTase, PMQRs and pAmpCs in CPKP strains collected during a 3-year-old period.

## Methods

### Bacterial isolates

Nanjing Drum Tower hospital is comprehensive tertiary hospital which manages 32 clinical departments, maintains 1460 patients, and admits more than 42,000 inpatients. A total of 108 non-replicate and consecutive *K. pneumoniae* isolates including 107 CRKP and 1 strains intermediate to carbapenem were collected from our hospital from January, 2012 to December, 2014. The Vitek 2 system (bioMérieux. Firenze, Italy) was used for isolate identification and antimicrobial drug susceptibility testing. Clinical specimens consisted of sputum (n = 44), blood (n = 18), urine (n = 12), secretion (n = 11), ascites (n = 9), bile (n = 6) catheter tips (n = 6), and others (n = 2). Among them, carbapenemase producers were further analyzed in this study. Clinical data such as the clinical features, laboratory results, and treatment were retrieved from the medical records department. The study protocol was approved by the Ethics Committee of Nanjing Drum Tower Hospital and written informed consent was obtained from all patients included in the study.

### Carba NP confirmatory test

For carbapenemase detection, Carba NP Confirmatory test was performed on 108 *K. pneumoniae* isolates according to the guidelines of Nordmann et al. [12] and CLSI 2015 [13]. Briefly, 2 microcentrifuge tubes for each patient isolate were labelled as “a” and “b” with 100 μL of bacterial protein extraction reagent (Tris–HCl 20 mmol/L lysis buffer) being added into each tube. A 1-μL loopful of tested bacteria from an overnight blood agar plate was emulsified in both tubes and vortexed for 5 s. Carba NP Solution A (2 mL 0.5 % phenol red solution and 180 μL 10 mM zinc sulfate solution were added into 16.6 mL clinical laboratory reagent water, pH 7.8 ± 0.1) was added into tube “a” and Carba NP Solution B (solution A + 6 mg/mL imipenem) into tube “b”, respectively. After the tubes were well vortexed, An incubation at 35 ± 2 °C for up to 2 h was needed. Test results were interpreted as follows: carbapenemase producers were reported when tube “b” became into light-orange, dark yellow, or yellow before 2 h, and tube “a” kept red or red–orange.

### ESBLs confirmatory testing

ESBL production was detected by using the 2015 CLSI recommendations for phenotypic confirmatory test [13]. In brief, disks containing 30 μg of cefotaxime and ceftazidime, either alone or coupled with 10 μg of clavulanate (Oxoid Ltd, Cambridge, UK), were placed at distances of 20 mm (center to center). When the inhibition zone differed by ≥5 mm, between at least 1 of the combination disks and its corresponding single antibiotic disk, the strain was identified as an ESBL producer. *E. coli* ATCC 25922 and *K. pneumoniae* ATCC 700603 were used as quality control strains.

### Detection of antimicrobial resistance determinants

DNA templates were prepared by the boiling method. All the 108 strains were screened for *bla*OXA-48 and *bla*NDM genes by PCR as previously described [14]. The 74 carbapenemase-producers identified by Carba NP Confirmatory test were further analyzed for carbapenemase encoding genes (*bla*KPC, *bla*IMI, *bla*SME, *bla*GES, *bla*VIM, *bla*IMP, *bla*NDM, *bla*GIM) by PCR-based techniques with subsequent sequencing according to the protocols as previously described [15], PMQRs (*qnrA*, *qnrB*, *qnrC*, *qnrD*, *qnrS*, *aac (6′)*-*Ib*-*cr*, and *qepA*) were detected by simplex PCR, followed by sequencing [16]. Among the 74 carbapenemase-producing strains, the presence of *bla*ESBLs (*bla*CTX, *bla*TEM, *bla*SHV) were checked for 42 strains positive for the ESBLs confirmatory testing according to the previous protocol [17], The 50 strains resistant to amikacin were further analyzed for the prevalence of 16S-RMTases (*armA*, *npmA*, *rmtA*, *rmtB*, *rmtC*, *rmtD*, and *rmtE*) [18] by multiplex PCR followed by single PCR for gene confirmation. Positive products were further purified with a DNA purification kit and then sent to the Majorbio Company (Shanghai, China) for sequencing. Sequences were analyzed by using the Chromas-Pro application and BLAST (http://www.ncbi.nlm.nih.gov/BLAST), and the subtypes of β-lactamase genes were confirmed by referring to the Lahey system (http://www.lahey.org/studies/).

### Multi-locus sequence typing

The sequence types of the 74 carbapenemase producers were determined by multi-locus sequence typing (MLST) analysis with 7 housekeeping genes including *gapA*, *infB*, *mdh*, *pgi*, *phoE*, *rpoB*, and *tonB* being amplified and sequenced according to Diancourt et al. [19]. Alleles and sequence types (STs) were assigned by using the MLST database (http://www.pasteur.fr/mlst/Kpneumoniae.html).

### Pulsed-field gel electrophoresis

Clonal relatedness of the 72 producing KPC-2 *K. pneumoniae* strains was analyzed by pulsed-field electrophoresis (PFGE) as previously described [20]. The restriction endonuclease XbaI (Fermentas, ABI, Germany) was used to digest the prepared genomic DNA and resultant DNA fragments were separated in a PFGE CHEF-DR III system (Bio-Rad Laboratories, Hercules, CA, USA) in 0.5× Tris–borate-EDTA buffer at 120 V for 19 h, with pulse times ranging from 2.2 to 54.2 s. The banding patterns were analyzed by the BioNumerics software (Applied Math, Sint-Maten-Latem, Belgium). Cutoff lines at 80 % were used to analyze genetic relatedness.

## Results

### Prevalence of carbapenemase and Antimicrobial susceptibility of CPKP isolates

Carba NP Confirmatory test showed that 74 out of 108 carbapenem resistant *K. pneumoniae* isolates were confirmed to be carbapenemase producers. PCR and molecular typing identified KPC-2 among 72 out of 74 carbapenemase producers and the other 2 isolates was found to produce NDM-1. Other carbapenemase-encoding genes were not detected. All the 74 carbapenemase producers found in our study were resistant to all of the β-lactams tested with 67.6 % (50/74) exhibiting resistance to amikacin and 72.73 % (54/74) exhibiting resistance to quinolones.

### Clinical characteristics of CPKP isolates

A total of 15 departments in our hospital have been involved with the clonal dissemination of KPC-2 producing *K*. *pneumoniae* isolates in our hospital with the intensive care units (ICU) being the commonest department (n = 21), followed by Emergency (n = 10) and Department of Neurosurgery (n = 9). Departments of Geriatrics and General Surgery, Infectious Diseases, Otolaryngology, Urology, Respiratory, Hematology, Neurology, Cardio-thoracic, and Cardiology have also been included. The isolates were mainly from the respiratory tract (n = 35), blood (n = 12) and urine (n = 8). Overall, the spread of KPC-2 producing *K. pneumoniae* ST11 clones co-carrying other resistance genes in hospital wards during 2012–2014 (Table 1) showed that compared to the data in 2012, such strains rapidly increased with more hospital wards being involved, especially in ICU. And a rapid decrease of such strains was observed during 2014 resulting from the strengthening implementation of hand hygiene in our hospital at the beginning of 2014 (personal communication). In addition, the Retrieval of NDM-producing *K*. *pneumoniae* strains showed that one strain was isolated from the blood of a 61-year-old male diagnosed as electrolyte imbalance after colorectal cancer surgery since he was admitted into ICU, persistent fever and blood culture showed severe septicemia caused by a NDM-producing *K*. *pneumoniae* strain, after a combination therapy of imipenem, tigecycline, and cefepime for 20 days, the patient died; the other NDM-producing *K*. *pneumoniae* was recovered from the secretion of a 41-year-old male who had undergone a serious burns on his whole body. No fever was observed although NDM-producing *K*. *pneumoniae* was isolated from the burn wounds of the patients. After a series of Rinse debridement treatment, the patient got a good wound healing and discharged.

**Table 1:** The spread of KPC-2 producing *K. pneumonia* ST11 clones associated with other resistance genes in hospital wards during 2012–2014

Isolation year	KPK-ST11	Antimicrobial Resistance determinants					Ward
		ESBLs	PMQRs	16S-RMTase	Carbapenemase	AmpC	
2012	10	8	10	7	10	0	ER(3), GS(2), NL(1), GA(1), NS(1), RP(1), VS(1)
2013	36	33	32	13	36	1	ICU(11), NS(6), ER(6), GS(3) GA(2), IDS(1), OG(1), UL(1), RP(1)
2014	11	8	9	1	11	1	ICU(4), CS(3), AD(1), GA(1), NS(1)

KPK-ST11: KPC-2 producing *K. pneumonia* ST11

*ER* emergency room, *ICU* Intensive Care Unit, *CS* cardiothoracic surgery, *NL* neurology, *AD* andrology, *UL* urology, *VS* vascular surgery, *NS* neurosurgery, *GS* general surgery, *GA* geriatrics, *RP* respiratory, *IDS* infectious diseases, *OG* Otolaryngology

### Prevalence of antimicrobial resistant determinants

ESBLs confirmatory testing showed that 42 strains were positive for ESBLs among the 74 carbapenemase-producing strains. PCR and DNA sequencing identified CTX-M, SHV, and TEM-1 type β-lactamases in 29 (39.2 %), 27 (36.5 %), 46 (62.2 %) isolates, respectively. CTX-M variants included 24 CTX-M-14, 3 CTX-M-15, 2 CTX-M-38; one SHV-1 and multiple SHV variants including SHV-11 (n = 2), SHV-12 (n = 13), SHV-28 (n = 2), SHV-31 (n = 6), SHV-160 (n = 2), and SHV-36a (n = 1) were observed. In addition, 2 DHA-1 were identified. 16S-RMTase including RmtB (n = 25) and Arm (n = 1) were identified among 26 out of the 50 carbapenemase-producers displaying resistance to amikacin. Fifty out of 74 carbapenemase producers were found to carry *oqxAB*. And the prevalence of both *oqxA* and *oqxB* detected in *K. pneumoniae* was high: 83.3 and 77.8 %, respectively. Of them, 48 *oqxAB*, 58 *oqxA* and 56 *oqxB* were detected among 69 carbapenemase producers highly resistant to fluoroquinolones. Whereas, 2 *oqxAB*, 2 *oqxA* and 2 *oqxB* were found among the 3 carbapenemase producers susceptible to fluoroquinolones.

In addition, one novel mutants named *oqxA11* and 3 novel *oqxB* mutants named *oqxB13*, *oqxB27*, and *oqxB28* were identified. And 3 kinds of *qnrB* variants including *qnrB6* (n = 1), *qnrB10* (n = 3) and *qnrB66* (n = 2) were identified. Other PMQRs were not detected.

Of particular concern, *qnrB6*, *oqxA*, *bla*TEM-1, and *bla*CTX-M-15 were identified in one NDM-producing isolate, albeit only *oqxB,* NDM-1 was found in the other one.

### MLST

**Fig. 1:**
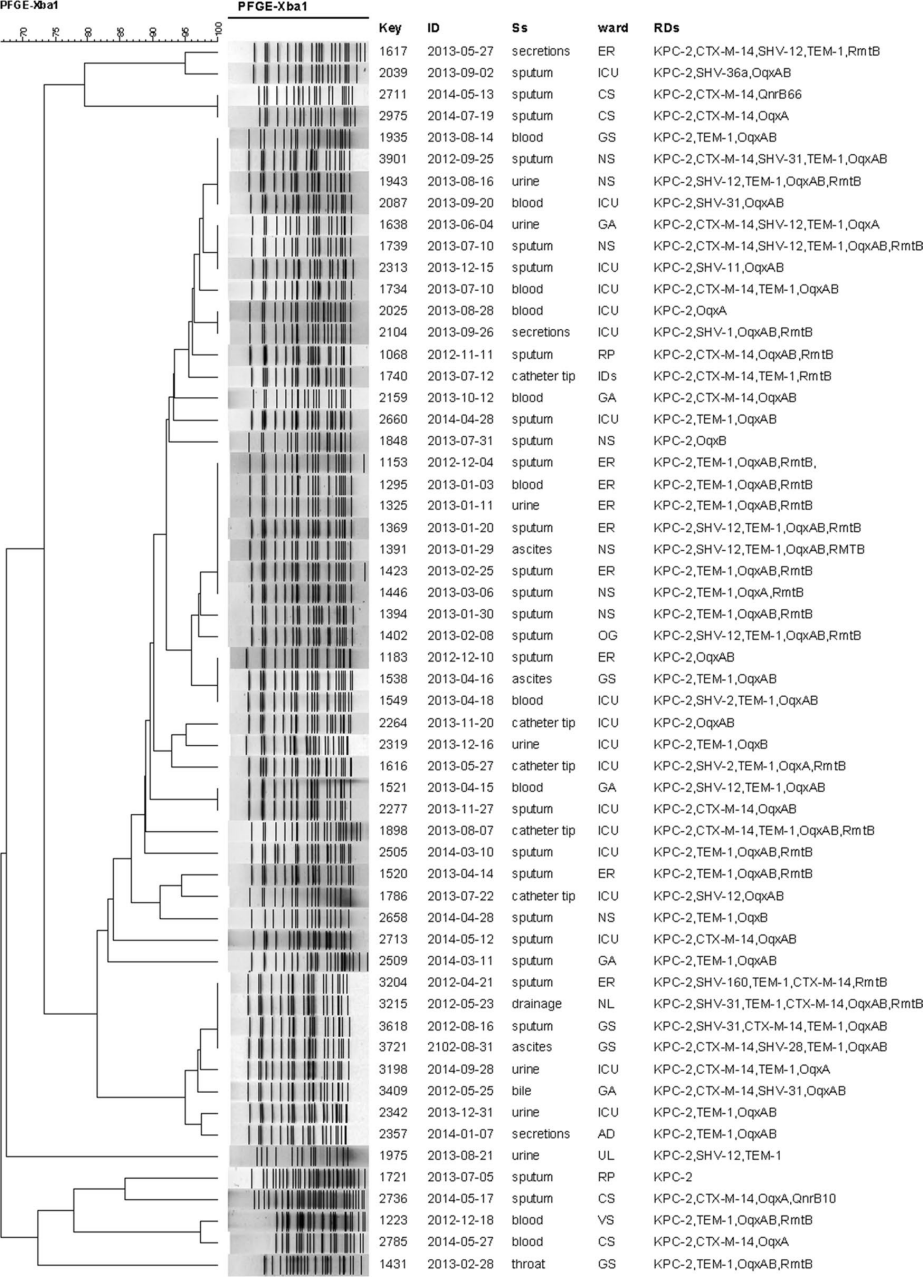
Dendrogram based on PFGE profiles of 57 KPC-2 producing *K. pneumoniae* ST11 isolates. The dendrogram was produced by the UPGMA algorithm based on the Dice similarity coefficient included five PFGE groups as defined based on 80 % similarity of PFGE profiles. *ID* isolation date, *Ss* samples, *RDs* resistant determinants, *ER* emergency room, *ICU* Intensive Care Unit, *CS* cardiothoracic surgery, *NL* neurology, *AD* andrology, *UL* urology, *VS* vascular surgery, *NS* neurosurgery, *GS* general surgery, *GA* geriatrics, *RP* respiratory, *IDS* infectious diseases, *OG* otolaryngology

MLST analysis of 74 carbapenemase producers revealed 10 different STs (Fig. 1). The most dominant ST was ST11 (78.4 %, 58/74), followed by ST15 (8.1 %, 6/74). The remaining isolates were typed as ST23, ST218, ST362, ST412, ST420, ST606, ST690, and ST709. MLST analysis of 2 NDM-1 producing *K*. *pneumoniae* isolates identified as ST826 and ST307, respectively.

### Clonal relatedness

**Fig. 2:**
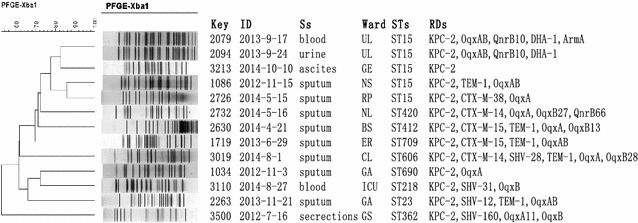
Dendrogram based on PFGE profiles of 13 KPC-2 producing *K. pneumoniae* isolates. The dendrogram was produced by the UPGMA algorithm based on the Dice similarity coefficient included five PFGE groups as defined based on 80 % similarity of PFGE profiles. *ID* isolation date, *Ss* samples, *RDs* resistant determinants, *ER* emergency room, *UL* urology, *GE* gastroenterology, *NS* neurosurgery, *RP* respiratory, NL neurology, *BS* burn surgery, *ER* emergency room, *CL* cardiology, *GA* geriatrics, *ICU* Intensive Care Unit, *GS* general surgery

According to the PFGE patterns of the isolates (Fig. 1), the 57 KPC-2 producing *K. pneumoniae* ST11 isolates exhibited 7 PFGE profiles. One major clonal groups composed of 47 closely related strains was observed among all the ST11 clones according to the 80 % similarity level. The PFGE data of the other 13 KPC-2 producing *K. pneumoniae* displayed a genetic diversity (Fig. 2). Among them, 2 PFGE profiles composed of 5 ST15 clones were observed. In addition, 2 strains including 1 ST11 clone and 1 ST15 one were non-typable by PFGE.

## Discussion

Clonal dissemination of CRKP in the health care settings frequently cause a potential threat to the public health with the increasing use of carbapenems in the clinical treatments worldwide. And we provided the first report on the clonal dissemination of *K*. *pneumoniae* ST11 with a high prevalence of OqxAB in a tertiary hospital in China.

In this study, 72 out of 74 carbapenemase producers carried KPC-2, indicating that KPC is the major CHBLs accounting for the carbapenem resistance and KPC-2 is the most prevalent enzyme which was in accordance with the previous studies both at home and abroad [3]. OqxAB, encoded by both chromosome and plasmids as a quinolone and olaquindox efflux pump, has become increasingly prevalent among members of *Enterobacteriaceae* over the past decade [21, 22]. It has been demonstrated that acquisition of the *oqxAB* encoding plasmids may facilitate the selection of CIP resistant *Salmonella typhimuriu* [23], and high expression of this pump contributes to reduced susceptibility to quinolones in clinical isolates of ESBL-producing *K. pneumoniae* [24]. A more recently published paper also described that overexpression of the *oqxAB* efflux pump in *K. pneumoniae* has been linked to an antibiotic cross-resistance phenotype [25] and OqxAB may contribute to tigecycline resistance in *K. pneumoniae* isolates [26]. Furthermore, a previous analysis of the genotypic and phenotypic characteristics of *K. pneumoniae* harboring *oqxAB*-like elements found that chromosomal *oqxAB* elements in *K. pneumoniae* strains exhibited cross resistance to olaquindox, chloramphenicol and the quinolones, whereas, transposition of the oqxAB operon from bacterial chromosome to plasmids results in more than 80-fold increase in the level of expression of the *oqxAB* pump, confirming that *oqxAB* efflux system is constitutively expressed when located in bacterial mobile elements [27]. The resistance mediated by the mutations in the quinolone-resistance determining regions in DNA gyrase and DNA topoisomerase IV of *K. pneumoniae* makes it difficult for us to speculate whether *oqxAB* in our study locates on plasmids or on the chromosome, and further study is needed to analyze the locations of these *oqxAB* operons and the role that *oqxAB*, *oqxA* and *oqxB* played in the fluoroquinolone development.

All together, the observed high prevalence of pump gene *oqxA* and *oqxB* in combination or alone in the KPC-2 producing *K. pneumoniae* ST11 represents a potential reservoir for the spread of these genes.

Of note, it’s the first time that we identified a new variant *qnrB66* as well as the novel mutant named *oqxA11* and 3 novel *oqxB* mutants named *oqxB13*, *oqxB27*, and *oqxB28* in *K. pneumoniae* which indicate that these PMQRs have been rapidly evolving under the selection pressure of antimicrobial agents in the healthcare settings.

Furthermore, the high prevalence of *rmtB* among KPC-2 producing *K. pneumoniae* isolates has been reported in China [28], it has also been characterized that *rmtB* and *bla*KPC-2 were located in the same plasmid and frequently disseminated among *K. pneumoniae* isolates under the selection pressure of antimicrobial agents [29].

Up to date, ST11 strains has been frequently reported to be the carriage of multiple resistant determinants such as CTX-M, KPC-2, as well as RmtB [8]. And the co-carriage of different ESCs (extended-spectrum cephalosporinaes) and 16S-RMTases, alone or in combination, were also noted among the KPC-2 isolates in our study, indicating that the clone dissemination of ST11 strain may carry different types of plasmids or more than one plasmid. Additionally, recent reports showed that ST11 is the dominant strain associated with carbapenem resistance in *K. pneumoniae* [30] and even also the predominant clone of tigecycline-resistant *K. pneumoniae* strains [26], indicating that *K. pneumoniae* ST11 has been developing as major high-risk clone. Taken together, these data indicated rapid evolution and expansion of the KPC-2 producing ST11 isolates with acquisition and spread of transferable resistance determinants in our hospital [31].

Noticeably, there is a potential dissemination of ST15 clone secondary to the ST11 clones in our study, indicating that ST15 is another major high-risk clones. This is consistent with a recent study [32]. ST23 has been frequently reported to be the carbapenem resistant strains [33], whereas, the other clones including ST218, ST362, ST412, ST420, ST606, ST690, and ST709 has not been reported to be involved with carbapenem resistance.

Of particular concern, NDM has been frequently found in *K. pneumoniae* isolates and involved with the healthcare associated outbreaks in China [34, 35] and the emergence of NDM-producing *K. pneumoniae* isolates in our hospital showed a spread potential of these strains and infection control measures should be emergently implemented to prevent the healthcare associated outbreaks since the choice of antimicrobial agents is quite limited once the patients were infected with NDM-producing *K. pneumoniae* isolates. Additionally, to the best of our knowledge, it’s the first time that we provide data on the *K. pneumoniae* ST826 and ST307 clones carrying NDM.

In recent years, the clonal dissemination of CRKP ST11 among Chinese hospitals has been frequently reported [32] and the wide distribution of the KPC-2-producing *K*. *pneumoniae* ST11 isolates co-carrying *oqxAB* and *rmtB* in our study indicate an intra- and inter- hospital cross-transmission of such strains in our hospital, which further strongly suggested that these isolates have a high potential to cause healthcare-associated outbreaks. Careful monitoring of carbapenem susceptibilities and rapid identification of colonized or infected patients as well as screening of carriers is quite necessary for implementation of infection control measures to prevent a scenario of endemicity.

## Abbreviations

CPKP: carbapenemase-producing *Klebsiella pneumoniae*
ESBLs: extended-spectrum β-lactamase
PMQRs: plasmid-mediated quinolone resistance genes
16S-RMTase: 16S rRNA methyltransferase
pAmpCs: plasmid-mediated AmpC enzyme
STs: sequence types
MLST: multi-locus sequence typing
PFGE: pulsed-field gel electrophoresis
ID: isolation date
Ss: samples
RDs: resistant determinants
ER: emergency room
ICU: Intensive Care Unit
CS: general surgery
NL: neurology
AD: andrology
UL: urology
VS: vascular surgery
NS: neurosurgery
GS: general surgery
GA: geriatrics
RP: respiratory
IDs: infectious diseases
OG: otolaryngology
GE: gastroenterology
BS: burn surgery
CL: cardiology
